# Greenness assessment of UPLC/MS/MS method for determination of two antihypertensive agents and their harmful impurities with ADME/TOX profile study

**DOI:** 10.1038/s41598-023-46636-5

**Published:** 2023-11-07

**Authors:** Hend M. Nagieb, Nada S. Abdelwahab, Maha M. Abdelrahman, Hala E. Zaazaa, Nermine S. Ghoniem

**Affiliations:** 1 Pharmaceutical Chemistry, Faculty of Pharmacy, Nahda University [NUB], Beni-Suef, 62511 Egypt; 2 Pharmaceutical Analytical Chemistry, Faculty of Pharmacy, Beni-Suef University, Alshaheed Shehata Ahmad Hegazy St, Beni-Suef, 62514 Egypt; 3 Analytical Chemistry, Faculty of Pharmacy, Cairo University, Cairo, Egypt

**Keywords:** Chemistry, Analytical chemistry, Green chemistry

## Abstract

Hypertension is described by the world health organization (WHO) as a serious medical problem that significantly affects the heart, brain and kidneys. It is a major cause of premature death worldwide. The present study aims to quantify the combination of captopril (CPL), hydrochlorothiazide (HCZ) and their harmful impurities; captopril disulphide (CDS), chlorothiaizde (CTZ) and salamide (SMD). *In-silico* study was conducted for estimation of pharmacokinetic parameters (ADMET) as well as toxicity profile of the proposed impurities. The results showed that the three impurities under investigation had poor permeability to CNS and cannot pass the blood–brain barrier (BBB), reducing the likelihood of causing side effects in the brain. On the other hand, all studied impurities were found to be hepatotoxic. In consequence, a highly sensitive and green ultra-performance liquid chromatography- tandem mass spectrometric (UPLC/MS/MS) method was developed and validated for separation of the cited drugs in the presence of their harmful impurities; methanol and 0.1% formic acid (90:10, v/v) mixture was used as a mobile phase, eluted at a constant flow rate of 0.7 mL/min at room temperature. Detection was adopted using a tandem mass spectrometer in a positive mode only for CPL and negative mode for HCZ, CDS, CTZ and SMD. Separation was performed within 1 min. Calibration graphs were found to be linear in the ranges of (50.0–500.0 ng mL^−1^), (20.0–500.0 ng mL^−1^), (10.0–250.0 ng mL^−1^), (5.0–250.0 ng mL^−1^) and (20.0–400.0 ng mL^−1^) corresponding to CPL, HCZ, CDS, CTZ and SMD, respectively. Additionally, comparative study of greenness profile was established for the proposed and reported methods using five green metric tools. The proposed method was found to be greener than the reported HPLC method. The developed (UPLC/MS/MS) method was validated according to (ICH) guidelines and it was found to has greater sensitivity, shorter analysis time and lower environmental impact compared to the reported methods.

## Introduction

**Figure 1:**
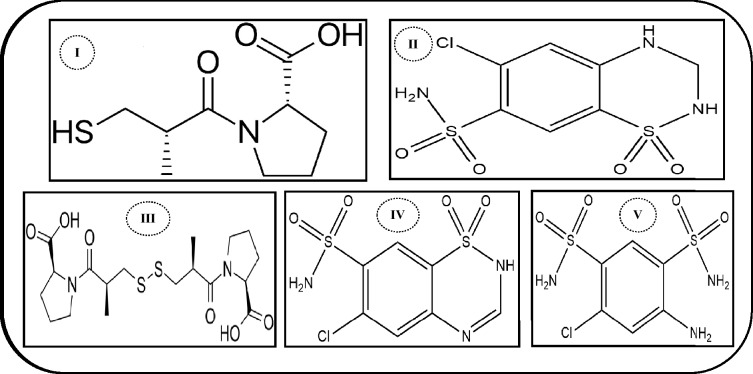
Chemical structures of (I) captopril, (II) hydrochlorothiazide; (III) capropril disulphide, (IV) chlorothiazide and (V) salamide.

Captopril (CPL) is chemically described as ((2S)-1-[(2S)-2-Methyl-3-sulfanylpropanoyl]pyrrolidine-2-carboxylic acid) as illustrated in Fig. 1^1^. It is an angiotensin converting enzyme inhibitor (ACEI) which acts by inhibition of angiotensin converting enzyme and consequently leads to vasodilatation and decreasing peripheral resistance so decreasing elevating blood pressure^2^. Hydrochlorothiazide (HCZ) is chemically known as (6-Chloro-3,4-dihydro-2H-1,2,4-benzothiadiazine-7-sulfonamide1,1dioxide) as described in Fig. 1^1^. It is a thiazide diuretic which acts by increasing sodium and water excretion which causes a decrease in extracellular volume; resulting in lowering blood pressure^3^. Combination of ACEI and diuretics was used to treat hypertension in patients whose pressure is not adequately controlled with monotherapy^3^.

Preserving human health is the main and ultimate goal. Presence of minute concentrations of harmful impurities may have had a negative effect on the efficacy and safety of the drug products and in consequence would affect seriously the human health. Therefore, the significance of quantifying the impurities with high sensitivity was originated. Analysis and quantification of active pharmaceutical ingredients in the presence of their stated pharmacopeial impurities is a difficult dilemma because of the high structure similarity which leads in consequence to similarity in physical and chemical properties making their separation and quantification a great challenge. Though the importance of quantifying the cited drugs in presence of their impurities in high sensitivity had originated.

HCZ has several impurities which are stated in British pharmacopoeia (B.P)^1^ including; Chlorothiazide (CTZ) (6-chloro-2H-1,2,4-benzothiadiazine-7-sulfonamide 1,1-dioxide) and salamide (SMD) (4-amino-6-chlorobenzene-1,3-disulfonamide) Fig. 1. The maximum accepted limit for both CTZ and SMD is 0.5%^1^. On the other hand, captopril disulphide is the dimer of CPL, specified to be its process related impurity and it is chemically (1,1¢-[disulfanediylbis[(2S)-2-methyl-1-oxopropane-3,1-diyl]] bis[(2S)-pyrrolidine-2- carboxylic] acid), Fig. 1. It is also considered as the oxidative product of CPL with a reported maximum specified limit of 1%^1, 4^.

*In-silico* study has proven to be effective in the last decades as it was found to be cost effective and time saving in the prediction of pharmacokinetic parameters and toxicity profile (ADMET) of the investigated impurities^5^. *In-silico* study and estimation of pharmacokinetic parameters (ADMET) was conducted for the three stated impurities and results revealed that the three impurities are hepatotoxic, contrary they are non-mutagenic and cannot cross blood–brain barrier (BBB).

Literature survey revealed many publications concerning analysis of HCZ and CPL either alone, in their combination or in combination with other drugs or with their impurities. The reported methods for their combined determination included UV-spectrophotometry^6^, chemiluminescence and artificial neural network^7^, HPLC^8, 9^ and voltammetric methods of analysis^10, 11^. For their determination in presence of other drugs, there are some methods including HPLC^12, 13^, UPLC/MS/MS^14^, GC-MS^15^ and by voltammetric method of analysis^16^. Only two approaches were described for determination of HCZ and CPL with their specified studied impurities; HPLC^17^ and capillary electrophoresis^18^*.*

Up till now, no reported LC-MS-MS method was described for quantitation of the studied drugs along with their chosen harmful impurities. All the reported methods lacked the sensitivity required to quantify the studied impurities with the concentration specified to detect their presence in minute amounts.

On the other hand, consuming hazardous chemicals and solvents or generating harmful wastes is considered a serious issue and it globally gains more attention. In consequence, green assessment of analytical methods is of great importance and it has been conducted by different methods. National environmental method index (NEMI) was the first applied qualitative greenness assessment tool^19^ with the advantage of being the simplest green metric tool. Its main drawback was its inability to cover a lot of GAC principles. The second tool was modified NEMI^20^. Although it was able to discuss more GAC principles as instrument energy and operator risk it still lacks consideration of some other important principles. The third green metric tool was Green analytical procedure index (GAPI)^21^. It is a one of qualitative greenness evaluation tool^20^. It is represented by pictograms with three coded colors (green–yellow and red). It covers more GAC principles than NEMI and modified NEMI green metric tools^20, 21^.

For Analytical eco-scale, it is considered a semi-quantitative tool taking into consideration more GAC principles than NEMI and modified NEMI such as occupational hazard, volume of consumed solvents, hazardous reagents, energy of instruments and amount of generated wastes^20, 22^.

Analytical Greenness (AGREE) is a quantitative green metric evaluation tool. It is described as a calculator for greenness of analytical methods and it covers the 12 principles of GAC. The (AGREE) calculator gives a numerical result which helps in easily evaluation of method greenness^23^.

The work in this manuscript aimed to develop a validated UPLC/MS/MS method to quantify CPL, HCZ and their harmful impurities with the highest possible sensitivity and shortest analysis time. Additionally, it aimed to predict the pharmacokinetics and toxicity profile of the chosen impurities using computer software. The greenness assessment of the method was evaluated by five metrics and then compared to those of the reported separation methods. The proposed method showed greater sensitivity, shorter analysis time and lower environmental impact compared to the reported methods. So, it can be used for quality control analysis during testing for the presence of such hepatotoxic impurities.

## Experimental

### Instruments

UPLC/MS/MS “Acquity Waters” 3100 “USA” was used for the chromatographic separation of the cited drugs, it was equipped with binary solvent manager, auto sampler and a tandem mass triple quadrupole detector. For CPL, the electrospray ionization (ESI) interface was operated on the positive mode while the negative mode was used for HCZ, CDS, CTZ and SMD. The multiple reaction monitoring (MRM) mode was operated and the Mass Lynx V4.1 software was used for processing the output signals, data acquisition and control hardware. This analysis was performed using Agilent poroshell 120EC-C18 (4.6 × 50 mm, 2.7 μm).

- Digital balance (Sartorius, German).
- Ultrasonicator Sonix TV SS-series (South Carolina, USA).
- pH meter (Jenway 3505, Staffordshire, UK).
- UV lamps with a short wavelength of 254 nm (Vilber Lourmat, Marne La Vallee, Cedex, France).

### Software and program

In silico ADME properties and toxicity profiling were implemented via Online pkCSM properties^24^ depending on 2D structural models, conducted via ChemBioDraw Ultra software (v 11.0), Cambridge Software Company, USA^25^.

### Materials and chemicals

#### Pure sample

Captopril and hydrochlorothiazide samples were kindly supplied from (Pharco Pharmaceuticals Co. Alexandria, Egypt). Their purity was tested by performing HPLC reported method^9^ and results were found to be 99.83% and 99.91%, respectively. CTZ and SMD standards were purchased from (Sigma-Aldrich Chemie GmbH, Germany) and their purity were tabulated to be 99.90% and 99.87%, respectively.

#### Pharmaceutical formulation

CAPOZIDE tablets 50/25 mg (Batch No. (J88C)) were claimed to contain 50 mg CPL and 25 mg HCZ and they were manufactured by (Squipp - Smithkline Beecham). The investigated CAPOZIDE tablets were purchased from local market.

#### Chemicals and solvents

- The consumed methanol, acetonitrile and formic acid were of UPLC/MS/ MS grade and they were purchased from (Sigma-Aldrich, Chemie GmbH, Germany).
- The used ethyl acetate was of HPLC grade (Fischer, UK). Iodine, glacial acetic acid and sodium thiosulphate were of analytical grade (Piochem for Chemical Co., Giza, Egypt). Water for injections (Otsuka, Pakistan Ltd).
- For following up the degradation of captopril: TLC aluminum plates F254 (20 × 20 cm) precoated with 0.25 mm Silica gel (Merck, Darmstadt,Germany).

### Solutions

#### Standard solutions

Stock solutions of all the investigated drugs and their impurities were prepared in concentration of (10 µg mL^−1^) by separately weighing 0.05 mg of each in 5 mL volumetric flask then dissolving the weighted powder in methanol and then completing the volume to the mark with the same solvent. Working solutions (1.0 µg mL^−1^) were prepared by suitable dilutions of the previously prepared stock solutions using methanol. Final dilutions for preparation of calibration standards, synthetic mixtures and validation standards were prepared in 5 mL calibrated flasks by further dilutions from working solutions using the same solvent.

#### Pharmaceutical formulation solution

Ten tablets of CAPOZIDE were accurately weighed and finely grinded. Amount equivalent to 0.5 mg CPL and 0.25 mg HCZ was weighed and then transferred into 5 mL volumetric flask, 3.0 mL methanol was added, ultra-sonicated for 10 min, filtered and the flask was completed to the mark with methanol to obtain sample solution of (100 µg mL^−1^). Further dilution of the prepared sample solution was then carried out using methanol to obtain sample working solution of (1.0 µg mL^−1^). Concentration within the linear range of the constructed calibration curve was then prepared in methanol and analyzed by the suggested method. Matrix effect was tested by application of standard addition technique on three different levels.

## Procedure

### Preparation of captopril disulphide (CDS)

It was prepared following the method published by (Moussa et al.^4^ and Rose^26^). 0.1 gm of CPL was weighed in a rounded flask and dissolved in 5 mL water and then I_2_ solution (0.05 M) was added dropwise to sample solution till appearance of faint yellow color. Excess I_2_ was then treated by addition of drops of sodium thiosulphate solution (0.1 M) till no yellow color^4, 26^.

The formed white precipitate was then filtered after washing with distilled water (3 × 15 mL). The separated precipitate was dried in oven at (40–45 °C) for one hour^4^. In order to ensure full degradation of CPL, TLC was performed using a stationary phase of TLC aluminum plates F254 (20 × 20 cm) precoated with 0.25 mm Silica gel and a developing system of ethyl acetate and acetic acid (12:1.2, v/v). The spots were visualized by exposing the plate to I_2_ vapors. The resulted degradation product was then characterized by the proposed method via detecting of its molecular weight.

### Chromatographic and mass spectrometric conditions

Analysis of the two drugs and their impurities and/ or oxidative degradation product were performed using isocratic elution of a mixture of methanol and 0.1% formic acid in proportion of (90 :10, v/v). The conducted mobile phase was eluted at flow rate of 0.7 mL/min for no more than 1 min at room temperature with 10 µL injection volume. Mass spectrometric conditions were set at: capillary voltage, 3000 v, ion source temperature, 150 °C; desolvation temperature, 400 °C. Cone voltages used for detection were 15, 35, 45 for CPL, HCZ and CTZ, respectively and 30 for both CDS and SMD; while for collision energies they were found to be 15, 20 and 30 for CPL, CDS and CTZ and 25 for both HC and SMD. For CPL, the electrospray ionization (ESI) interface was operated on the positive mode while the negative mode was used for HCZ, CDS, CTZ and SMD. Multiple reaction monitoring mode was operated, detection of molecular weight transitions for the investigated drugs was conducted and the used molecular weight transitions were: 218.08 to 115.39 for CPL, 295.93 to 204.48 for HCZ, 431.00 to 181.88 for CDS, 293.92 to 213.84 for CTZ and 283.94 to 135.97 for SMD.

### Preparation of calibration standards

For construction of calibration curves; aliquots covered the linear range of (50.0–500.0 ng mL^−1^), (20.0–500.0 ng mL^−1^), (10.0–250.0 ng mL^−1^), (5.0–250.0 ng mL^−1^) and (20.0–400.0 ng mL^−1^) corresponding to CPL, HCZ, CDS, CTZ and SMD, respectively were accurately measured and transferred into series of 5 mL volumetric flask then volume was completed to the mark using methanol. The prepared samples were injected in the UPLC/MS/MS system and analyzed using the previously mentioned chromatographic conditions. The calibration curves were established for the cited components by plotting the peak area for each after dividing by constant concentration of the targeted analyte versus the concentration in (ng mL^−1^). Linear regression equations were then calculated.

### Application of the method to pharmaceutical formulation

Concentrations within the linearity range of each drug were prepared from the previously prepared pharmaceutical dosage form working solution (1.0 µg mL^−1^). Samples were then analyzed following the method under chromatographic conditions. Concentration was then calculated from the equations of calibration curve previously counted. Samples of standard addition technique was also prepared and analyzed by the developing method and amounts of spiked drugs were obtained.

### Application of validation parameters

Validation parameters as accuracy, precision, LOD and LOQ were conducted according to the ICH guidelines. Regarding accuracy, three samples in triplicates over three concentration levels were prepared, analyzed and average % recovery was then calculated. Standard addition was performed to confirm the method accuracy. It was conducted by choosing three different concentrations at different levels and added to the dosage form. The standard addition solutions were then analyzed in triplicates. It was expressed as the (average recovery % ± SD). Specificity was confirmed by testing the separation between the resolved peaks using the system suitability parameters. Method precision was performed at two levels; repeatability and intermediate level. Repeatability was performed by preparing three different concentrations covering three different levels and analyzed in triplicate in the same day. While for intermediate precision the three selected concentration levels were prepared and analyzed three times in three consecutive days. Precision was expressed as (RSD%). Limits of detection and quantitation were determined through the following mathematical equations: LOD = (SD of the response/slope) × 3.3; LOQ = (SD of the response/slope) × 10. The standard deviation (SD) of the response was calculated from SD of intercepts of three regression lines of three calibration graphs over three concentration levels.

## Results and discussion

Analysis of pharmaceutical compounds in the presence of their process impurities is a serious issue as it has a direct impact on human health due to the possible expected toxicity and side effects of such impurities. Structure similarity between drugs and their specified impurities results in high similarity in their physical and chemical properties. In consequence, the separation of such drugs in the presence of their impurities is characterized by great difficulty. On the other hand, due to possible toxicity or side effects of possibly encountered impurities, the need for a specific and selective analytical method has become a top priority. The new developed method had the goal of quantifying the investigated impurities and make confirmation that it is in the accepted reference limits. This has been achieved by using the UPLC/MS/MS hyphenation technique. In the presented work, a highly sensitive, selective, precise and green UPLC/MS/MS method was developed and validated for simultaneous determination of HCZ and CPL and their toxic impurities; CTZ, SMD and CDS. Pharmacokinetic and toxicity of the cited impurities was conducted by application of ADMET/TOX profile study. Moreover, greenness profile was evaluated using five green metric tools NEMI, modified NEMI, GAPI, analytical eco-scale and AGREE. Beside that the proposed method was evaluated as a green method but also it has the advantages of high sensitivity especially for the studied toxic impurities.

### Confirmation of oxidative product of captopril (Caprtopril disulphide)

**Figure 2:**
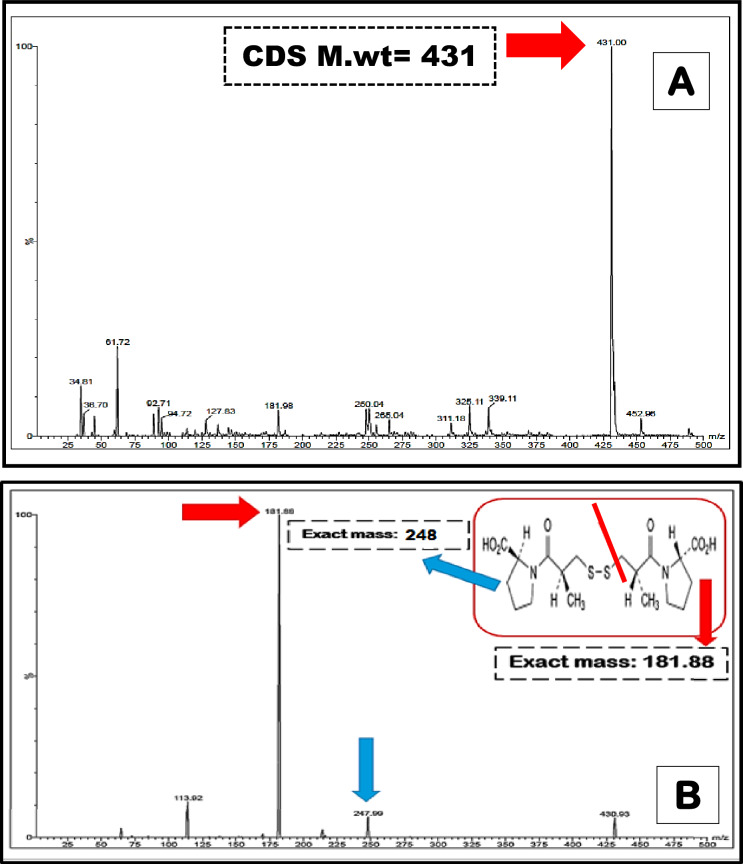
Mass spectrum of precursor and product ion of captopril disulphide representing molecular weight of captopril disulphide (**A**) fragmentation and product ion (**B**).

The structure of the previously prepared oxidative product; captopril disulphide (CDS) was confirmed by the produced mass spectrum. It was found to be the dimer of CPL which is formed by coupling two thiol groups (-SH) of two CPL molecules and thus was confirmed by the base peak of molecular weight equal to 431, Fig. 2.

### Prediction of the pharmacokinetic properties and toxicological properties using ADMET

One of the most important goal of scientific research in the field of pharmaceutical industries is not only the ability to determine the proportions and quantities of official impurities, but also the capability to predict the pharmacokinetic behavior within the body to help illustrate its potential effect on human health. With the great and rapid boom in the computer world, it has become possible to predict these pharmacokinetic properties with the click of a button and through a single session on the computer. There are now many programs that perform this task in a few minutes^24^, which contributes significantly and effectively to saving time, effort, and money. By implementing one of these programs^24^ to the current study, it was noticed that the cited impurities under investigation has a bad effect on the liver. Hence the importance of the current study to develop rapid and precise method to quantify the impurities and drugs with high sensitivity.

This passage describes the use of pkCSM software to conduct *in-silico* ADMET studies, which predict the pharmacokinetic and toxicological properties of impurities and oxidative products in antihypertensive medications. The software estimates various parameters such as water solubility, intestinal absorption, clearance, the ability to cross the blood–brain barrier, effects on the CNS, hepatotoxicity, and mutagenicity. The results showed that CDS has low water solubility and intestinal absorption, which may decrease its bioavailability. The three impurities under investigation had poor permeability to CNS and cannot pass the blood–brain barrier (BBB), reducing the likelihood of causing side effects in the brain. Captopril disulphide (CDS) had a high total body clearance, while CTZ and SMD had low clearance rates. The toxicological properties of the impurities were also tested, and all studied impurities were found to be hepatotoxic^5^. The passage emphasizes the need for developing sensitive and selective analytical methods to quantify impurities in antihypertensive medications in the presence of their official hepatotoxic impurities. Such methods will help to ensure that impurities fall within accepted reference limits and reducing the undesired toxicity and side effects, results were listed in Table 1.

**Table 1:** ADMET properties of the investigated impurities; chlorothiazide, salamide and captopril disulphide.

	Items	CDS	CTZ	SMD	Reference*^5^
Absorption	Water solubility (log mol/L)	− **2.054**	− 3.478	− 3.111	Solubility increased by decreasing log S
	CaCO-2 permeability (log Papp in 10^−6^ cm/ S)	− 0.085	− 0.008	− 0.026	High permeability > 0.90
	Intestinal absorption (%)	**24.072**	79.694	71.079	High absorbed > 30%
	P-Glycoprotein substrate	NO	NO	NO	
Distribution	VDss (log L/Kg)	− 1.091	− 0.669	− 0.699	Low ˂ − 0.15 High > 0.45
	Fraction unbound (Fu)	0.405	0.523	0.623	
	BBB permeability (log BB)	− **1.716**	− **1.063**	− **1.126**	Log BB ˂ − 1 poorly distributed to the brain Log BB > 0.3 cross the BBB
	CNS permeability (log PS)	− **3.271**	− **3.187**	− **3.250**	Log PS ˂ − 3 unable to penetrate CNS Log PS > − 2 penetrate CNS
Metabolism	CYP2D6 substrate	YES	NO	NO	This can be positively correlated to the lipophilicity of the compound to metabolism related toxicity
	CYP3A4 substrate	YES	NO	NO	
	CYP1A2 inhibitor	NO	NO	NO	
	CYP2C19 inhibitor	NO	NO	NO	
	CYP2C9 inhibitor	NO	NO	NO	
Excretion	Total clearance (log mL/min/Kg)	**0.329**	− **0.017**	− **0.117**	
	Renal OCT2 substrate	NO	NO	NO	
Toxicity	Max. tolerated dose (log mg/kg/ day)	− 0.19	0.645	1.19	Low ≤ 0.477 High > 0.477
	Oral rat acute toxicity (LD50) (mol/kg)	2.018	2.691	1.754	
	AMES test	Non − mutagen	Non − mutagen	Non − mutagen	Mutagenic or not
	Hepatotoxicity	**YES**	**YES**	**YES**	
	T. pyriformis toxicity (log μg/L)	0.285	0.367	0.173	Not toxic ˂ − 0.5 Toxic > − 0.5
	Minnow toxicity (log mM)	1.864	2.04	2.424	Highly acute toxic ˂ − 0.3 Not highly acute toxic > − 0.3

Significance values are in bold.

*Reference values of the pKCSM pharmacokinetics predictions properties.

### Method optimization

#### Mass spectrometric and chromatographic conditions

**Figure 3:**
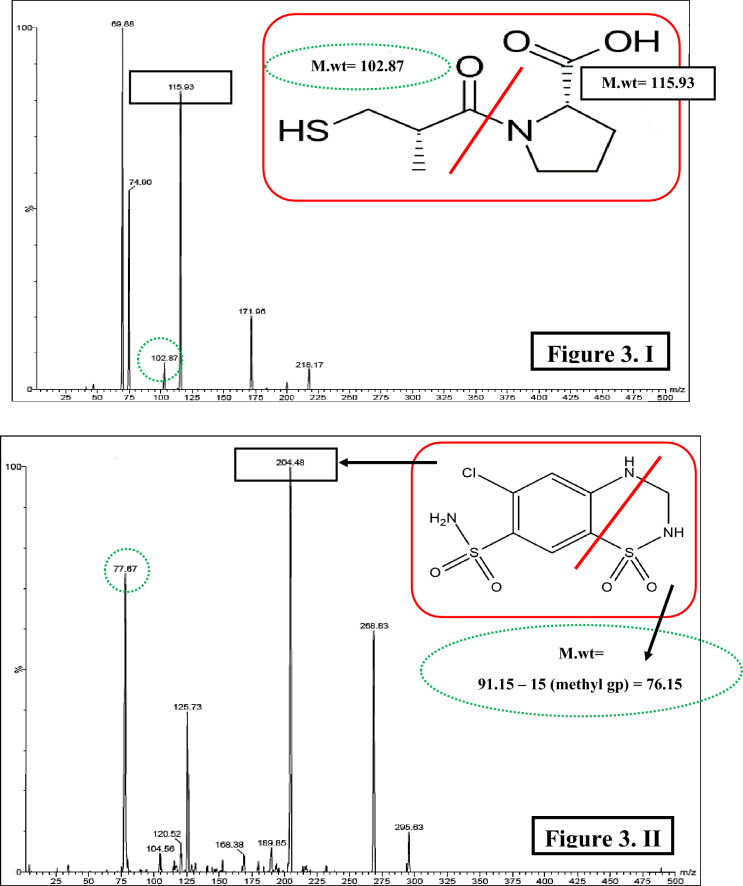

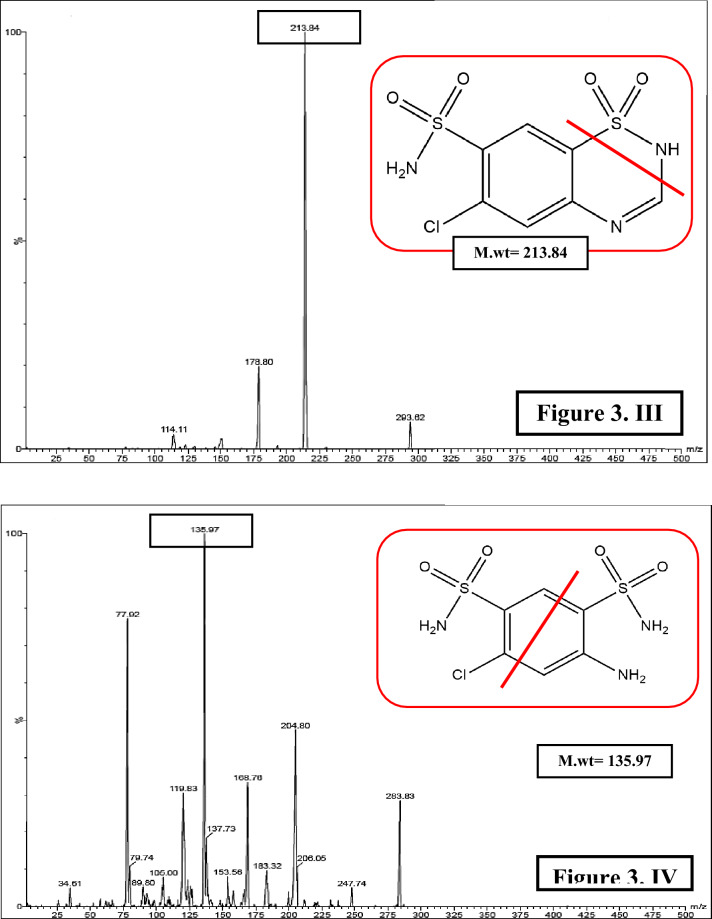
Chemical structures and product ion spectra of captopril (*m/z* 218.08 → 115.93) (I), hydrochlorothiazide (*m/z* 295.93 → 204.48) (II), chlorothiazide (*m/z* 293.92 → 213.84) (III) and salamide (*m/z* 283.94 → 135.97) (IV).

Optimization of the proposed method was divided into two phases; starting with the first phase which will discuss optimization of mass spectrometric conditions with the aim of obtaining the best intensities and shapes for the resolved peaks. Each of the cited compounds was analyzed separately and several conditions were adapted to get the best intensities. Electrospray ionization (ESI) mode was operated in the positive mode for CPL only while negative mode conducted good peaks and intensities for other investigated compounds. The precursor ions for CPL, HCZ, CDS, CTZ and SMD were found to be 218.08, 295.93, 431, 293.92 and 283.94, respectively. In accordance to obtain optimum sensitivity; parameters such as collision energy, cone volt, capillary voltage and ion source temperature were optimized. Consequently, the following daughters (product ions) were selected as they were the most stable fragments which were found to be 115.39, 204.48, 181.88, 213.84 and 135.97 for CPL, HCZ, CDS, CTZ and SMD, respectively, Fig. 3. All mass spectrometric optimized parameters were summarized in Table 2.

**Table 2:** Optimized mass spectrometric conditions for the simultaneous determination of hydrochlorothiazide and captopril and their impurities.

Drug	Retention time	M.Wt	MRM transition	Ion mode	Cone voltage (V)	Collision energy (eV)	Dwell time (ms)
CPL	0.80	218.08	115.93	+ve	15	15	28
HCZ	0.73	295.93	204.48	−ve	35	25	28
CDS	0.82	431.00	181.88	−ve	30	20	28
CTZ	0.72	293.92	213.84	−ve	45	30	28
SMD	0.74	283.94	135.97	−ve	30	25	28

The second phase includes optimization of different chromatographic conditions such as mobile phase composition and flow rate those have significant effects on resolution, sensitivity and selectivity of the method.

**Figure 4:**
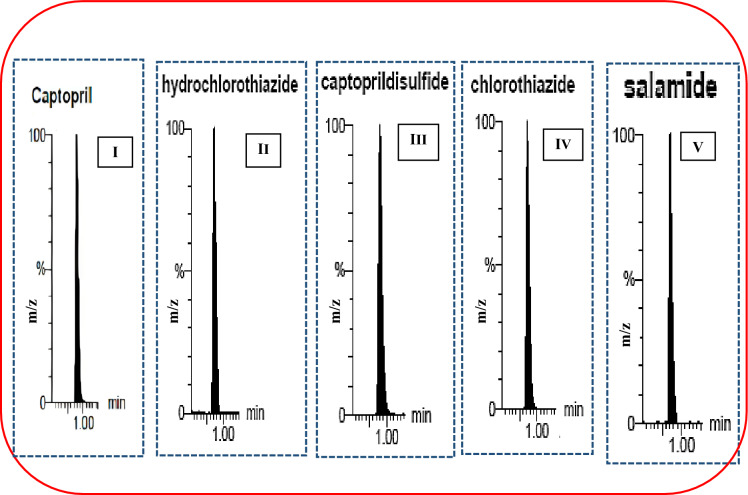
Representative multiple reaction monitoring chromatograms of captopril (I), hydrochlorothiazide (II), captopril disulphide (III), Chlorothiazide (IV) and salamide (V).

At first, acetonitrile was tested in different ratios (80–90%), poor results were obtained regarding peak symmetry and sharpness and this has been translated in the form of broad and forked peaks. Different ratios of methanol were then tested and it was found that it significantly enhanced the chromatographic resolution. First trials started with 80% methanol, resulting in slight improvement in tailing of peaks. In the framework of the endeavor to improve the peak sharpness and tailing to increase the sensitivity of the method, the percentage of methanol was increased up to 95% and the ratio 90% was selected which resulted in the optimum peaks shape and separation. Additionally, mobile phase flow rate was tested, starting with flow rate of 0.3 mL/min, where the peak tailing was clearly obvious. By increasing the flow rate gradually up to 0.7 mL/min, complete improvement in the peaks tailing for all components was resulted. Consequently, the best chromatographic conditions for separation of the investigated mixture was; isocratic elution of methanol and 0.1% formic acid (90 :10, v/v) at 0.7 mL/min within a time frame not more than 1 min. This chromatographic condition led to separation of the studied drugs in the following retention time: 0.8, 0.73, 0.82, 0.72 and 0.74 for CPL, HCZ, CDS, CTZ and SMD, respectively, Fig. 4. All mass spectrometric optimum parameters and retention time for resolved peaks were summarized in Table 2.

### Method validation results

According to ICH guidelines^27^, validation parameters like linearity, accuracy, specificity, selectivity, LOD and LOQ were evaluated and results were summarized in Table 3.

**Table 3:** Analytical and regression parameters for analysis of captopril and hydrochlorothiazide and their impurities by the proposed UPLC-MS method.

Parameters	CPL	HCZ	CDS	CTZ	SMD
Calibration range (ng mL^−1^)	50–500	20–500	10–250	5–250	20–400
Correlation coefficient (r)	0.9999	0.9999	0.9999	0.9999	0.9999
Intercept	− 0.0780	0.0452	0.0368	0.0290	0.1176
Slope	0.0108	0.0096	0.0097	0.0099	0.0088
Accuracy (%)^a^	99.53	100.04	100.69	99.69	99.46
Repeatability (RSD %)^b^ (Intraday precision)	0.034	0.030	0.019	0.074	0.015
Intermediate precision (RSD %)^c^	0.044	0.067	0.036	0.100	0.040
Limit of detection (LOD, ng mL^−1^)^d^	15.68	5.25	2.84	1.28	5.73
Limit of quantification (LOQ, ng mL^−1^)^d^	47.52	15.91	8.61	3.87	17.36

^a^Average % recovery of analysis of three samples in triplicates over three concentration levels.

^b^The intraday precision (n = 3), average of three different concentrations repeated three times within one day (100, 150, 300 ng mL^−1^) for CPL and HCZ, and (100, 150, 200 ng mL^−1^) for CDS, CTZ and SMD.

^c^The interday precision (n = 3), average of three different concentrations repeated three times on three successive days (100, 150, 300 ng mL^−1^) for CPL and HCZ, and (100, 150, 200 ng mL^−1^) for CDS, CTZ and SMD.

^d^Limits of detection and quantitation were determined via calculations LOD = (SD of the response/slope) × 3.3; LOQ = (SD of the response/slope) × 10.

*Where SD of the response was calculated from SD of intercepts of three regression lines of three calibration graphs over three concentration levels.

#### Linearity

Linearity of the established method was tested, the computed linear regression equations for the calibration curves revealed excellent linearity with perfect correlation coefficient (r) ˃ 0.999.

#### Range

The investigated drugs revealed linearity covering the following concentration ranges: (50–500 ng mL^−1^), (20–500 ng mL^−1^), (10–250 ng mL^−1^), (5–250 ng mL^−1^) and (20–400 ng mL^−1^) corresponding to CPL, HCZ, CDS, CTZ and SMD, respectively, each concentration was analyzed three times (N = 3) Table 3. Calibration curves were constructed, linear regression equations were calculated and the corresponding recovery percent were obtained. Good correlation coefficient (r) were obtained (> 0.999) confirming linearity of the method within the working range.

#### Accuracy

Accuracy was expressed as recovery % and it was calculated after analysis of three samples in triplicates (N = 3) over three concentration levels. Results were found to be 99.53, 100.04, 100.69, 99.69 and 99.46% corresponding to CPL, HCZ, CDS, CTZ and SMD, respectively. From the previous result it was found that the recovery % was close to 100% indicating high accuracy of the developed method as the results were in agreement with the accepted limits and were summarized in Table 3.

#### Specificity

Well defined and highly resolved peaks shown in Fig. 4 assured specificity of the proposed method. It was furtherly assured by application of the proposed method to the pharmaceutical formulation (CAPOZIDE). Also, the recovery % of the cited drugs CPL and HCZ in the pharmaceutical formulation were (102.19 and 101.54%) and it was found to be in the accepted limits (90–110%) indicating high method specificity. Results in Table 1 S revealed no interference from encountered excipients or impurities.

#### Precision

According to ICH guidelines; precision could be expressed at three levels; Repeatability, intermediate and reproducibility. Repeatability is the first level representing the variations within short time interval under the same operating conditions. It could be calculated by analyzing three different concentrations three times (N = 3) within the same day which was found to be 0.034, 0.030, 0.019, 0.074 and 0.015 for CPL, HCZ, CDS, CTZ and SMD, respectively.

The second level termed intermediate precision which represent the occurred variations due to random events as performing the experiments along longer periods of time e.g. analyzing three different concentrations three times within three successive days (N = 3) which was found to be 0.044, 0.067, 0.036, 0.100 and 0.040 for CPL, HCZ, CDS, CTZ and SMD, respectively. Precision was expressed in term of percent relative standard deviation (RSD %) and the lower (RSD %) value, the more precise the method is. From the repeatability and intermediate precision results in Table 3 it was assured that the suggested method is precise due to the very low value of the (RSD %).

#### LOD and LOQ

Limits of detection and quantitation were determined and found to be 15.68, 5.25, 2.84, 1.28 and 5.73 ng mL^−1^ while LOQ were 47.52, 15.91, 8.61, 3.87 and 17.36 ng mL^−1^ for CPL, HCZ, CDS, CTZ and SMD, respectively. Value of LOD and LOQ helps assure the method sensitivity. The lower the LOD and LOQ values the higher the sensitivity. From the tabulated results, one can conclude that the developed method has the sensitivity required to quantify the lowest amount of the studied impurities due to the low LOD and LOQ values expressed in ng/mL, Table 3.

##### Statistical comparison

The previously calculated results conducted by the proposed method were statistically compared with reported HPLC method^9^ and results were presented as *F*- and Student’s *t*- tests. *F*- test were found to be 2.17 and 3.51 but Student’s *t*- test were 0.63 and 1.77 for CPL and HCZ, respectively. Calculated results indicated that there was no fundamental difference between the calculated and tabulated one. The calculated values were less than the tabulated ones at probability of 5% which indicating no significance difference between the developed and the reported HPLC one, results are summarized in Table 1 S.

### Greenness profile

For developing a green analytical method, the selection of solvents with minimum environmental impact as well as minimum amounts is a pivotal step. Accordingly, the UPLC chromatographic system was chosen because of its advantage of consuming little amounts of solvents. Thus in consequence led to decreasing the produced amount of wastes.

In this context, great diversity of green assessment tools has been developed and varied between qualitative tools as NEMI, modified NEMI and GAPI, semi-quantitative like analytical eco-scale and quantitative such as AGREE^20^. A comparative study between the proposed UPLC/MS/MS method and other reported separation methods was also done^9, 17, 18^.

The first and oldest applied green metric was NEMI^19^. It is considered a qualitative method characterized by simplicity and ease of performance. The main drawback was that it does not cover and discuss all 12 principles of GAC. NEMI could not differentiate between the developed UPLC/MS/MS and the reported ones. Regarding the proposed method and reported methods^9, 18^, first and second quadrant were blank due to the usage of hazardous solvents which were listed in official lists, Table 4. While the reported method^17^ had a three blank quadrants due to its high amount of waste for separation (57.5 mL + 100 mL for column equilibration).

**Table 4:** Green assessment and comparison between the proposed and reported methods.

Analytical methods	Proposed UPLC MS/MS method	I- Reported method^18^	II- Reported method^9^	III- Reported method^17^
NEMI				
Modified NEMI				
GAPI				
Eco-scale	**80**	**82**	77	71
AGREE				
Calibration range for CPL and HCZ (µg mL^−1^)	**CPL: 0.05–0.5**	CPL: 2400–4800	CPL: 20–200	CPL: 5000–10,000
	**HCZ: 0.02–0.5**	HCZ: 1200–2400	HCZ: 10–100	HCZ: 2500–50,000
Run time (Min.)	**1**	3	5	20
Quantitative ranges of impurities (µg mL^−1^)	CTZ: 0.005–0.25 SMD: 0.02–0.4 CDS: 0.01–0.25	CTZ: 2–20 SMD: 0.6–20 CDS: 4–40 HCZ impurity C: 1.6–20	Impurities were not determined	CTZ: – SMD: 2.5–5 CDS: 30–60
In-silico study	√	–	–	–
Green assessment and comparison study	√	–	–	–

Reported method I: is capillary electrophoresis for determination of CPL and HCZ in presence of CDS, CTZ, SMD and HCZ impurity C in 3 min. New capillaries were flushed with 1 M NaoH for 5 min, followed by 0.1 M NaoH and water for 5 min each. Before every run, the capillaries were conditioned by flushing with methanol for 2 min, 0.1 M NaoH for 2 min, water for1min and BGE for 3 min. The selected working conditions: BGE, 100 mM borate buffer pH 8.55 (8.48–8.62), 64 mM (60–68 mM) sodium cholate, 6.1% v/v (5.4–6.8% v/v) n-butanol, 12 mM (11–13 mM) γ-CD. Voltage, 27 kV (26–28 kV), temperature, 21 °C and measured current was about 85 μA. Separations were carried out in a fused-silica capillary (50 mm inner diameter, 375 mm outer diameter, total length 33.0 cm) with a detection window at 24.5 cm. Solvent consumption calculated by capillary electrophoresis calculator program.

Reported method II: is HPLC for determination of CPL and HCZ using methanol: water (45:55, v/v), as the mobile phase pumped at flow rate 1 mL/min for 5 min. with UV detection at 210 nm.

Reported method III: is HPLC for determination of CPL and HCZ in presence of CDS and SMD using methanol: 0.05% aqueous phosphoric acid (25: 75, v/v) pumped at 2 mL/min for 8 min. the flow rate was increased to 4.5 mL/min for the next 7 min. and the methanol was increased to 45%, then methanol ratio was decreased to 25% at flow rate 2 mL/min. for the last 5 min.

The second assessment tool is modified NEMI^20^. The most noticeable change was the assessment of instrument energy and operator risk. The proposed UPLC/MS/MS method has more yellow triangle in comparison with the reported HPLC method^9, 18^ which was obviously represented in its pentagram. This was due to consuming formic acid which was listed in NFPA as health hazard and its high energy consumption due to usage of mass spectrophotometric detector, Table 4. For the reported method^17^, has four yellow triangle because of the massive solvent consumption as previously mentioned under NEMI green assessment tool.

For green analytical procedure index (GAPI) metric tool^21^, it is considered one of qualitative metric tool taking into consideration more GAC principles than other qualitative tools. GAPI tool has the advantages of being simple and easily implemented, could evaluate the entire analytical methodology starting with sample collection to final determination. For the proposed method it had four red zones only in the GAPI pictograms while for the reported capillary electrophoresis method^18^ it had five red zones. On the other hand, the proposed method had seven green zones in contrast with the capillary electrophoresis reported method^18^ which had eight green zones. This is due to that the developed method consumes more energy than the reported one, represented in Table 4. While, for the other two reported methods in the comparison^9, 17^ they had more red and yellow zones with less green zones because of consuming larger amount of solvents.

Analytical eco-scale^20, 22^ is a semi-quantitative tool taking into consideration more GAC principles than NEMI and modified NEMI. It was able to differentiate between the compared methods as it took in consideration the amount and hazardous of each solvent singly. The maximum eco-score is (100) for ideal green method. The proposed UPLC/MS/MS method and reported methods^9, 18^ had eco-score ˃75 which interpreted as excellent green methods. The reported LC^18^ has the highest eco-score = 82 which almost equal the score of the proposed UPLC/MS/MS with score = 80 which are the more green methods than the reported^9^ with eco-score = 77. While, for the reported method^17^ with eco-score = 71 which is just acceptably green^28^ as it recorded eco-score between 50 and 75.

For the proposed UPLC/MS/MS method, despite of generating the lowest amount of waste (< 1 mL) as a result of using ultra-performance liquid chromatography, the used mass spectrometric detector led to increasing the energy per sample along with using formic acid (of higher pictogram than phosphoric acid). While, for the reported method^18^, despite that capillary electrophoresis consumed lower energy per sample than LC methods but the method used solvents of danger signal word with high number of pictograms as n-butanol Table 2 S. Looking at the eco-scale of reported method^17^, it is obvious that it has the lowest score in comparisone with the proposed and other reported methods due to using of massive amounts of solvents with high penality points. From the foregoing, it could be concluded that eco-scale as a semi-quantitative tool could differentiate between the proposed and reported method from greenness point of view.

AGREE is a quantitative green metric evaluation tool that could be described as a calculator for greenness of analytical methods^23^. The closer we get to 1, the segment's color turn to dark green, contrary by getting close to zero its color will turn to red. In AGREE, the 12 GAC principles were covered and evaluated. From results summarized in Table 4, it was noticed that proposed UPLC/MS/MS and the reported capillary electrophoresis method^18^ had almost the same scores of 0.64 and 0.69, respectively. Results proved that they are greener than the other comparable reported LC methods^9, 17^ ( AGREE score of 0.59 and 0.49, respectively). The reported capillary electrophoresis method^18^ had the privilege of producing the lowest amount of waste and consuming the lowest energy. In contrary the proposed method has shorter run time which allows analyzing higher number of samples per hour and most of consumed solvents are bio-based resulting in increasing in the AGREE score to become close to the other method. Regarding reported method^17^ it had the lowest score due to consumption of large amount of hazardous solvents as well as long run time. 

Considering performance of the method, the proposed UPLC/MS/MS has many advantages over all reported separation methods as it is highly selective and the most sensitive one. It could selectively separate and quantify the specified impurities up to the limit stated in the British pharmacopoeia^1^. Also, the developed method characterized by the lowest run time (1 min.) leading to decreasing amount of consumable solvents.

Moving on to another positive point regarding the developed method, which is that it is an environmentally friendly method and aims to use the least amount of organic solvents that have a bad impact on the environment and thus negatively affect human health. There is no doubt that the use of small and limited quantities of solvents leads to the production of very small quantities of waste that are harmful to the environment. Therefore, this advantage of the developed method avoids the disadvantage of consuming high amounts of energy as a result of using LC/MS/MS instrument in contrast to capillary electrophoresis one^18^. Despite that the developed method had high energy consumption but the run time was very small (1 min.). This led to analyzing large number of samples per hour so the energy consumption will not be great relative to other used instruments. This led to that the developed method recorded the highest Eco- score and AGREE values after the capillary electrophoresis method^18^ and the results were found very close to each other. One can conclude that the developed method is environmentally friendly one. Comparison between the proposed and the reported methods are presented in, Table 4.

## Conclusion

Accurate, precise, selective and highly sensitive green UPLC/MS/MS method was developed for simultaneous analysis of CPL, HCZ and their harmful process impurities CTZ, SMD and CPL oxidative product and impurity (CDS). Pharmacokinetic parameters for impurities under investigation were conducted by application of ADMET study which revealed hepatotoxicity and T. pyriformis toxicity of the studied impurities. A comparative study of greenness profile of the developed method and some reported ones was done and results proved the low negative environmental impact of the investigated method. Using UPLC in analysis results in obvious decrease in analysis run time and amount of waste generated. Hyphenation of UPLC with MS/MS as a detector contributed to increasing sensitivity of the proposed method which makes sense with the aim of the work about determination of encountered harmful impurities within their limited pharmacopoeial concentration levels.

### Supplementary Information

Supplementary Tables.

## Data Availability

All data generated or analyzed during this study are included in this published article (and its Supplementary Information files).
