# Supplementary material for: Greenness assessment of UPLC/MS/MS method for determination of two antihypertensive agents and their harmful impurities with ADME/TOX profile study
Source: Sci Rep. 2023 Nov 7;13:19318. doi: 10.1038/s41598-023-46636-5 (PMC10630503; doi:10.1038/s41598-023-46636-5)
Supplement: Supplementary file 1 — Supplementary Tables. [file 41598_2023_46636_MOESM1_ESM.docx]

**Table 1 S:** Statistical comparison of the results obtained by the proposed UPLC-MS method and the reported HPLC method for the analysis of Capozide ^®^ tablets.

| **Captopril** | | | |
| --- | --- | --- | --- |
| **Parameters** | **UPLC/MS/MS** | **Reported method ^b^** [9] | |
| **Mean (%)^a^** | 102.19^*^ | 102.08 | |
| **SD** | 0.250 | 0.370 | |
| ***n*** | 6 | 6 | |
| **Student’s *t* test (2.228)^c^** | 0.63 | | |
| ***F* test (5.505)^c^** | 2.17 | | |
| **Standard addition technique** | | | |
| **Parameters** | **Taken (ng/mL)** | | **Recovery %** |
|  | 50 | | 99.63 |
|  | 100 | | 99.14 |
|  | 150 | | 100.22 |
| **Mean± SD** | 99.66 ± 0.54 | | |
| **Hydrochlorothiazide** | | | |
| **Parameters** | | | |
| **Mean (%)^a^** | 101.54^*^ | 101.30 | |
| **SD** | 0.160 | 0.300 | |
| ***n*** | 6 | 6 | |
| **Student’s *t* test (2.228)^c^** | 1.77 | | |
| ***F* test (5.505)^c^** | 3.51 | | |
| **Standard addition technique** | | | |
| **Parameters** | **Taken (ng/mL)** | | **Recovery %** |
|  | 50 | | 99.22 |
|  | 200 | | 100.28 |
|  | 250 | | 100.21 |
| **Mean± SD** | 99.90 **±** 0.59 | | |

^a Average percentage recoveries of six determinations.^

^b Reported method is HPLC for determination of CPL and HCZ using methanol: water (45:55, v/v), as the mobile phase with UV detection at 210 nm.^

^c Figures between parentheses represent the corresponding tabulated values of t and F at^ *^p^* ^= 0.05.^

^* The concentration of CPL and HCZ estimated in dosage form is 300 and 150 ng/mL, respectively.^

**Table 2 S:** Eco-scale scores of the developed and reported methods.

| **Parameters** | **Proposed UPLC/MS/MS** | pp | **I -Reported method** [18] | pp | **II- Reported method** [9] | pp | **III- Reported method** [17] | pp |
| --- | --- | --- | --- | --- | --- | --- | --- | --- |
| **PP of solvent = subtotal**  **PP × number**  **of pictogram × signal**  **word**  **Consumed volume**  **= run**  **time × flow**  **rate × solvent percentage**  **in system** | Deionized Water  Methanol  Consumed volume  = 5.63 mL  Subtotal PP = 1 [solvent  < 10 mL]  Signal word = 2  Danger [more severe  hazard = 2]  No. of pictogram = 3  Formic acid  Consumed volume  = 0.00007 mL  Subtotal PP = 1 [solvent  < 10 mL]  Signal word = 2  Danger [more severe  hazard = 2]  No. of pictogram = 3 | 0  6  6 | **^*^ Flushing capillaries:**  Sodium hydroxide  Consumed volume  = 0.0015 mL  Subtotal PP = 1 [solvent  < 10 mL]  Signal word = 2  Danger [more severe  hazard = 2]  No. of pictogram = 1  Methanol  Consumed volume  = 0.0000314 mL  Subtotal PP = 1 [solvent  < 10 mL]  Signal word = 2  Danger [more severe  hazard = 2]  No. of pictogram = 3  Deionized water  **^*^ Working conditions:**  Sodium cholate  Consumed volume  < 1 mL [Subtotal PP = 1 [solvent  < 10 mL]  Signal word = 0  [No signal word]  No. of pictogram = 0  n-butanol  Consumed volume  < 1 mL [Subtotal PP = 1 [solvent  < 10 mL]  Signal word = 2  Danger [more severe  hazard = 2]  No. of pictogram = 3  Deionized water  ᵧ-cyclodextrin  Consumed volume  < 1 mL [Subtotal PP = 1 [solvent  < 10 mL]  Signal word = 0  [No signal word]  No. of pictogram = 0 | 2  6  0  0  6  0  0 | Deionized Water  Methanol  Consumed volume  = 12.025 mL  Subtotal PP = 2 [solvent  10-100 mL]  Signal word = 2  Danger [more severe  hazard = 2]  No. of pictogram = 3  Phosphoric acid  Consumed volume  = 2 mL  Subtotal PP = 1 [solvent  < 10 mL]  Signal word = 2  Danger [more severe  hazard = 2]  No. of pictogram = 1 | 0  12  2 | Deionized Water  Methanol  Consumed volume  = 20.675 mobile phase + 33.33 column conditioning + 65 mL sample preparation= 119.005 mL  Subtotal PP = 3 [solvent  ˃100 mL]  Signal word = 2  Danger [more severe  hazard = 2]  No. of pictogram = 3  Phosphoric acid  Consumed volume  = 0.0184125 mobile phase + 0.33 column conditioning= 0.3484125 mL  Subtotal PP = 1 [solvent  < 10 mL]  Signal word = 2  No. of pictogram = 1 | 0  18  2 |
| Energy | > 1.5 kWh per sample | 2 | ≤ 0.1 kWh per sample | 0 | ≤ 1.5 kWh per sample | 1 | ≤ 1.5 kWh per sample | 1 |
| Occupational hazard | Analytical process  hermetization | 0 | Analytical process  hermetization | 0 | Analytical process  hermetization | 0 | Analytical process  hermetization | 0 |
| Wastes | 1–10 mL    No treatment | 3  3 | < 1  No treatment | 1  3 | ˃ 10 mL  No treatment | 5  3 | ˃ 10 mL  No treatment | 5  3 |
| Total penalty points | **20** | | **18** | | **23** | | **29** | |
| Analytical Eco-Scale  total score | **80** | | **82** | | **77** | | **71** | |
